# Off-Court Generic Perceptual-Cognitive Training in Elite Volleyball Athletes: Task-Specific Effects and Levels of Transfer

**DOI:** 10.3389/fpsyg.2019.01599

**Published:** 2019-07-24

**Authors:** Marie-Therese Fleddermann, Holger Heppe, Karen Zentgraf

**Affiliations:** ^1^ Department of Movement Science and Training in Sports, Institute of Sport Sciences, Goethe-University Frankfurt, Frankfurt, Germany; ^2^ Department of Human Performance and Training in Sports, Institute of Sport and Exercise Sciences, University of Münster, Münster, Germany; ^3^ Otto Creutzfeldt Center for Cognitive and Behavioral Neuroscience, University of Münster, Münster, Germany

**Keywords:** training intervention, perceptual-cognitive expertise, multiple-object tracking, skill transfer, elite athletes

## Abstract

**Background:** The nature of perceptual-cognitive expertise in interactive sports has gained more and more scientific interest over the last two decades. Research to understand *how* this expertise can be developed has not been addressed profoundly yet. In approaches to study this with interventional designs, only few studies have scrutinized several levels of transfer such as to the field. Therefore, the aim of this study was to examine the efficacy of a generic off-court perceptual-cognitive training in elite volleyball players on three different levels: task-specific, near-transfer, and far-transfer effects. Based on overlapping cognitive processes between training and testing, we hypothesized task-specific improvements as well as positive near- and far-transfer effects after a multiple-object tracking training intervention.

**Methods:** Twenty-two volleyball experts completed a 8-week three-dimensional (3D) multiple-object tracking (3D-MOT) training intervention. A control group (*n* = 21; volleyball experts also) participated in regular ball practice only. Before and after training, both groups performed tests on the 3D-MOT, four near-transfer tests in cognitive domains, and a far-transfer, lab-based, and volleyball-specific blocking test.

**Results:** The results of the 2 × 2 analysis of variance (ANOVA) (group, time) showed significant interaction effects in the 3D-MOT task [*F*(1,40) = 93.10; *p* < 0.001; $\eta_{p}^{2}$ = 0.70] and in two near-transfer tests [sustained attention: *F*(1,40) = 15.45; *p* < 0.001; $\eta_{p}^{2}$ = 0.28; processing speed: *F*(1,40) = 12.15; *p* = 0.001; $\eta_{p}^{2}$ = 0.23]. No significant interaction effects were found in the far-transfer volleyball test.

**Conclusions**: Our study suggests positive effects in task-specific and two near-transfer tests of a perceptual-cognitive intervention in elite volleyball athletes. This supports a partial overlap in cognitive processing between practice and tests with the result of positive near-transfer. However, there are no significant effects in far-transfer testing. Although these current results are promising, it is still unclear how far-transfer effects of a generic perceptual-cognitive training intervention can be assured.

## Introduction

In elite sports, the optimization of different aspects of performance has progressed extensively (e.g., Williams and Ericsson, 2005; Yarrow et al., 2009). One aspect of interactive sport games such as soccer, basketball, or hockey that has gained scientific interest in recent years refers to perceptual-cognitive expertise, which is the ability of an athlete to scan and process the environment and integrate relevant information into existing knowledge coupled with the execution of adequate (motor) responses (Marteniuk, 1976; Mann et al., 2007; Broadbent et al., 2015). Therefore, it is necessary for an athlete’s success to interact in quickly changing environments including teammates, opponents, referees, coaches, and ball movements and to select and execute adequate actions. For example, during a game, (beach-)volleyball athletes process visual-tactical requirements such as monitoring the ball and teammates’ and opponents’ trajectories, and based on this, select a motor action that will facilitate the greatest offensive or defensive advantage (Lennartsson et al., 2015; Fleddermann and Zentgraf, 2018). This is not a sequential and independent process, but an interaction between many aspects such as divided attention, decision-making, and motor behavior embedded in a tactical context (Raab, 2014).

Perceptual-cognitive skills have mostly been studied under two different approaches: the expert performance approach and the cognitive component skill approach. Studies based on the *expert performance approach* investigated the perceptual-cognitive expertise of an athlete by using sports-related stimuli in a sport-specific context, e.g., measuring decision-making, attention, or memory skills in simulated sport-specific settings or in a valid ecological sport context. Results of these studies show that elite athletes perform faster and more accurately compared to nonathletes or semiathletes (for a meta-analysis, see Mann et al., 2007). For example, Abernethy (1990) showed an expert advantage in a squash anticipation task when participants were asked for the judgment of stroke direction based on early kinematic information. Ripoll et al. (1995) showed advantages for elite athletes in decision-making and visual search strategy task in boxing. Alternatively, studies using a *cognitive component skill approach* examined fundamental perceptual-cognitive skills per se in a sport-unspecific context and investigated the relationship between sport expertise and fundamental perceptual-cognitive skills. Results of these studies indicate that elite athletes perform better than nonathletes in fundamental cognitive tests such as processing speed or varied attention paradigms (for a meta-analysis, see Voss et al., 2010). These positive findings mainly refer to interactive or team sports such as tennis or volleyball and less so to static sports (e.g., swimming). It was therefore suggested that interactive (team) sports require high levels of cognitive and perceptual functions such as processing speed, working memory, decision-making, or executive functions (for a review, see Walton et al., 2018) and that the related skills are practiced as a side effect in their regular training settings. Additionally, a number of recent studies further corroborate a significant relation between fundamental cognitive functions and sport expertise especially in interactive team sports, e.g., in executive tests in soccer (Vestberg et al., 2012; Verburgh et al., 2014) or in executive control tasks and visuospatial attentional processing task in volleyball (Alves et al., 2013). These two approaches appear to be relevant in elucidating the link between expertise and superior perceptual cognitive skills in sports.

As many studies have shown the importance of perceptual-cognitive expertise (Furley and Wood, 2016) and a relationship between sports expertise and different perceptual-cognitive skills, there is an increasing interest in the development and training of the latter. Studies investigated the effects of perceptual-cognitive training (PCT) in different paradigms such as quiet eye training (for an overview, see Vine et al., 2014). Hadlow et al. (2018) classified this in three different categories: sports vision training (SVT), which uses generic stimuli for developing visual skills; PCT, which uses sport-specific stimuli (images and videos) for developing perceptual-cognitive skills; or modified perceptual training (MPT), which uses specific on- or off-field techniques for developing athletes’ perceptual skills. Most of these studies focused on task-specific practice effects in sport beginners or semi-elite athletes. For example, Murgia et al. (2014) showed improvements of anticipatory skills (i.e., predicting the direction of penalty kicks) after a 8-week perceptual intervention training in skilled soccer goalkeepers. Schorer et al. (2018) demonstrated benefits in retention after a 4-week, PCT intervention in young soccer players. However, in elite sports, there are only few studies that investigated the development of perceptual-cognitive expertise *via* PCT interventions (for overviews, see Zentgraf et al. 2017; Hadlow et al., 2018). Especially in this domain, the aim to maximize every aspect of performance is strong. Mostly, perceptual-cognitive skills in team sports are thought to be improved by regular game-like ball practice *per se*. However, a perceptual-cognitive off-court intervention with transfer of these capacities to the field might support the development of perceptual-cognitive team sport skills. In a systematic review addressing exactly this issue, Zentgraf et al. (2017) examined the effectiveness of PCT in elite sports and only found 16 training studies in this domain. For example, Faubert (2013) showed task-specific practice effects in different team sport athletes such as soccer, rugby, and ice hockey players after 15 sessions in the three-dimensional multiple-object tracking (3D-MOT). Moreover, Gabbett et al. (2007) examined the effects of a video-based temporal occlusion decision-making training in elite softball players and found improvements in decision accuracy and no effects in decision time. Hohmann et al. (2016) investigated the effects of a decision-making training in national handball candidates and found practice improvements in decision time and best-action accuracy.

One claim is that not only task-specific practice effects should be considered but also the extent to which a transfer to other domains and to the field takes place. Transfer effects refer to those skills which are not directly trained. These could manifest themselves in similar tasks (near-transfer) or in game-like situations (far-transfer). Indeed, some studies investigated the efficiency and transfer benefits of cognitive training interventions. This mainly applies to studies from other research areas and less so to the sport context. PCT intervention studies in children (Alloway and Alloway, 2013; Dovis et al., 2015), adults (Karbach and Kray, 2009; Schmiedek et al., 2010; Shipsted et al., 2012; Karbach and Verhaeghen, 2014), or the elderly (Smith-Ray et al., 2013) supported the malleability of cognitive skills; specific improvements were convincingly shown in trained tasks and—to a lesser extent but still to a small to medium size—transfer effects in other measures. Parsons et al. (2016) studied task-specific practice and near-transfer effects in students based on a 5-week PCT with the 3D-MOT task. They found improvements in the 3D-MOT task (task specific) as well as in working memory, attention, and processing speed (near-transfer). Thus, transfer effects, which could be based on overlapping, or similar cognitive processes and networks (Dahlin et al., 2008; Voss et al., 2010) are shown relatively consistently.

As an extended level of transfer, studies also investigated further-transfer or as one of the most important levels far-transfer (for overviews, see Hadlow et al. 2018; Harris et al., 2018; Walton et al., 2018) using ecological valid situations. Furthermore, Hadlow et al. (2018) described in their review that the far-transfer level should be addressed in a field-based test, which is similar to a real-life game or a competitive situation. Further, they identified three factors (targeted perceptual function, stimulus correspondence, and response correspondence) that may influence transfer to the field. In other domains, Legault and Faubert (2012) employed a 3D-MOT training in the elderly and found improvements in biological motion perception, indicating further-transfer evidence in dynamic (real-life) scenes. Simons et al. (2016) also suggested evidence for far-transfer in everyday activities. In the sports domain, there are only some studies that examined transfer effects. For example, Ducrocq et al. (2016, 2017) suggested transfer effects of an adaptive working memory training resulting in performance improvements in an untrained near-transfer test and also in a field tennis test under pressure (far-transfer). Also, Hüttermann and Memmert (2018) demonstrated positive effects in the athlete’s attentional window after a 10-week varied lab-based training including different training tools such as the Stroop or cueing task in individual and team athletes compared to an active control group. Further, Broadbent et al. (2017) used a video-based sport-specific anticipation training in tennis and found improvements in the field-based transfer test. In contrast to these findings, Abernethy and Wood (2001) did not find transfer effects after a 4-week combined visual and motor performance training. They concluded that PCT programs are not transferable to sports, and the results show the lack of evidence to enhance on-field performance (Hadlow et al., 2018).

In elite sports, Romeas et al. (2016) examined passing, dribbling, and shooting accuracy in soccer players after an off-court multiple object training (over 5 weeks) and found weak to moderate improvements in passing accuracy (established by coaches). They concluded that training in processing complex and dynamic scenes could improve an athlete’s performance on the field. Balasaheb and Sandhu (2008) also showed improvements in a visual-task and in batting performance in real-game situations after a 6-week PCT intervention. However, overall, there are only few studies measuring different levels of transfer–especially in elite sports. Based on the overall aim to maximize performance in the field, Zentgraf et al. (2017) differentiated intervention effects on different levels of transfer in elite sports using four different categories: (1) task-specific practice effects, (2) near-transfer practice effects (e.g., similar cognitive tasks), (3) further-transfer effects (e.g., sensorimotor sport skills), and (4) far-transfer effects (e.g., game-like competition performance). The results showed that most of the 16 studies addressed task-specific practice effects (93%), but only few studies addressed the transfer to near (19%), further (42%), or far (19%) domains. Especially in elite sports, the far-transfer level represents the most important level with the open question of what reflects a meaningful improvement in the field. Also, Hadlow et al. (2018) defined transfer into the field as the most important transfer and suggested a (far) transfer test, which provides dynamic, goal-directed tasks in a competitive sport-specific context. Therefore, one aspect of far-transfer effects could relate to superior multitasking capacities in elite athletes. Fleddermann and Zentgraf (2018) showed cognitive-motor interference in elite athletes in a volleyball-specific game-like blocking task. Dual-task costs occurred in a highly automated volleyball-specific blocking task when a second perceptual-cognitive task (e.g., decision-making, divided attention) was added. Jumping performance (e.g., jumping height and the length of the first step after ready-block position) decreased under these dual-task condition compared to a single-task condition. Results of this study indicate an overlap between visual processing requirements for motor performance and for tracking game parameters in the dual-task situation. Based on the multiple resource model of Wickens (2002), the authors concluded limited multiple resources in the dual tasks, which lead to a decreased jumping performance. Consequently, one benefit of PCT could be to enhance athletes’ individual capacities. For example, performance in elite sports can be maximized through the reduction of interference effects between game-oriented perceptual-cognitive load and motor preparation/execution *via* interventions.

Based on the above, the aim of the study was to examine the efficacy of an additional off-court, visually based PCT in young elite volleyball players on three different levels: (1) task-specific practice level, (2) near-transfer level, and (3) far-transfer level. In the light of previous findings (Faubert, 2013; Parsons et al., 2016; Romeas et al., 2016), we hypothesized task-specific practice improvements as well as positive transfer effects (near/far) after an 8-week PCT (3D-MOT) compared to a control group, which only participates in systematic and regular ball practice, with controlled overall practice time in both groups. Further, based on the complex structure of the task, we used the 3D-MOT task, which relates to the perceptual-cognitive demands in dynamic sport situations (Faubert and Sidebottom, 2012). The targeted perceptual skills were general visual attention, awareness, and working memory (Hadlow et al., 2018). Previous literature moreover (Faubert, 2013; Parsons et al., 2016; Romeas et al., 2016) indicated task-specific effects and also near- and far-transfer effects. Based on overlapping cognitive processes between the practice task and other cognitive skills, we expected improvements for the intervention group in similar cognitive near-transfer tasks (processing speed, memory span, sustained attention, and working speed) and far-transfer effects in a lab-based, sport-specific game situation (volleyball blocking task in a single task and two dual tasks with perceptual-cognitive demands).

## Materials and Methods

### Participants

Fifty-one (inter)national (beach-)volleyball athletes volunteered for this study. Eight of them were excluded due to several reasons (retirement, injury, and break-off), so 43 athletes finished the pre- and posttest and were included in this study. All of the 43 players had elite senior or elite junior status, were members of national volleyball teams on the senior or junior level, or played in the 1st to 3rd division in Germany. All athletes had ball practice at least four and up to eight times a week during the study. Participants were recruited from a German volleyball talent development center, a first-league volleyball club, and other higher-league volleyball clubs in the region. Twenty-two athletes were included in the intervention group (two males, mean age = 16.38, SD = 1.7), and 21 formed the active control group (five males, mean age = 21.38, SD = 4.53). Participants (and their parents/legal guardians if players were under 18) gave written informed consent prior to any data collection, and the study protocol was approved by the ethics committee of the faculty of psychology and sport science, University of Münster.

### Procedure

The intervention group completed–in addition to regular ball and athletic practice–a specific dynamic PCT with the 3D-MOT task. The training intervention lasted 8 weeks with two units per week, each lasting 30 min. A unit comprised three sessions, 8 min each, with a 3-min rest in between. The first session was a baseline measurement and highly standardized. In the second and third parts of each training session, we added gross motor tasks to combine the perceptual-cognitive task with a motor task. Athletes executed a volleyball-specific motor task simultaneously to the 3D-MOT task (e.g., block jumps, setting over the head, tossing, or other ball control drills) or a volleyball-unspecific action (e.g., skipping). All training sessions were carried out in the lab and guided by a test conductor. The control group progressed with their regular practice, such as ball and athletic practice.

In a pre-post design, both groups were tested on a training-specific task, near-transfer tasks, and a far-transfer task. The training-specific task consisted of two baseline measurements in the 3D-MOT task. The near-transfer tests included four cognitive tests related to sustained attention (d2-R), memory span (KAI-N), working speed (KAI-N), and processing speed [Zahlenverbindungstest (ZVT)]. To measure far-transfer effects, a volleyball-specific decision-making task in the motor behavior lab was set up (see below for details).

### Dependent Variables and Data Analysis

#### Task-Specific Practice Test: 3D-Mot

The 3D-MOT task with the NeuroTracker™ Core Program by CogniSens Athletics Inc. from the University of Montreal was used on a computer for the pretests, posttests, and training interventions. The setting of the 3D-MOT measurements followed the setting of Faubert (2013).

Participants stood in an angle of 46° in front of the 3D compatible 60″ TV screen wearing 3D glasses and observed eight spheres in a 3D domain. First, four of the eight spheres changed their color for 1 s from yellow to orange. Participants were instructed to memorize these spheres. In a second step, for 8 s, all spheres moved randomly through the 3D domain with a specific velocity. After 8 s, the spheres stopped and the participants were asked to indicate the four “orange” spheres (targets). Then, participants got feedback, and the next trial started. Overall, one session lasted 8 min and consisted of 20 trials. The subjects gave their answers verbally, and the experimenter recorded the answers on a keyboard. In order to define their individual level, the velocity of the spheres adapted automatically after each trial following a staircase procedure. The first trial started at a given speed. If the athlete identified all spheres correctly, the speed of the next trial was faster. If the athlete did not identify all spheres, the speed of the next trial was slower (Faubert and Sidebottom, 2012). At the end of each session, the program calculated an individual speed threshold for each session. The higher the speed threshold, the better the performance.

In the pre- and posttest, participants completed two sessions. The dependent variable was the mean of the individual speed thresholds in two sessions. To define the performance improvements over the eight training weeks, participants completed a baseline session at the beginning of each training. This session was highly standardized. Afterward, to couple the perceptual-cognitive task with motor actions, the subjects performed two sessions with additional volleyball-specific (e.g., block jumps, ball drills) or volleyball-unspecific (e.g., tapping) tasks.

#### Near-Transfer Tasks (Cognitive Tests)

**Processing speed:** Processing speed was measured by the ZVT (Oswald and Roth, 1987). In this paper-and-pencil test, participants must connect the numbers from 1 to 90 in the correct order. Overall, the test consisted of four test sheets. The dependent variable was the arithmetic mean of the processing time in the four different sheets in seconds. The less time needed, the better the performance.

**Memory span:** Memory span was measured by a subtest of the KAI-N (Lehrl and Blaha, 2001). In this test, which is in German, the test conductor speaks out a sequence of letters or numbers, and the participants must repeat this sequence in the correct order. The sequence is extended after each correct repetition and ends when the subject is unable to repeat the sequence. Memory span is calculated by using the number of correct repetitions of letters and numbers. The higher the score, the better the performance.

**Working speed:** Working speed was measured by the letter readout test, which is a subtest of the KAI-N (Lehrl and Blaha, 2001). In this subtest, which is in German, participants must read out a randomized sequence of letters as quickly as they can. In total, they must read four sequences with a short rest in between. The dependent variable is the fastest reading time in seconds.

**Sustained attention:** Sustained attention was measured by the d2-R (Brickenkamp et al., 2010). This paper-and-pencil test measures attention and concentration ability under time pressure. The test consists of 14 lines with 47 characters per line with a randomized order of the letters “d” and “p.” Each letter is equipped with one, two, three, or four vertical strikes below or above the letter. The task of the participants is to mark the letter “d” with two stripes. All other characters are distractors, and participants are supposed to ignore these characters. For each line, participants have 20 s, and there is no break between the lines. The dependent variable is CP, which is calculated from the processed target objects and the errors. The higher the score, the better the performance.

#### Far-Transfer Tasks (Volleyball-Specific Test)

**Figure 1:**
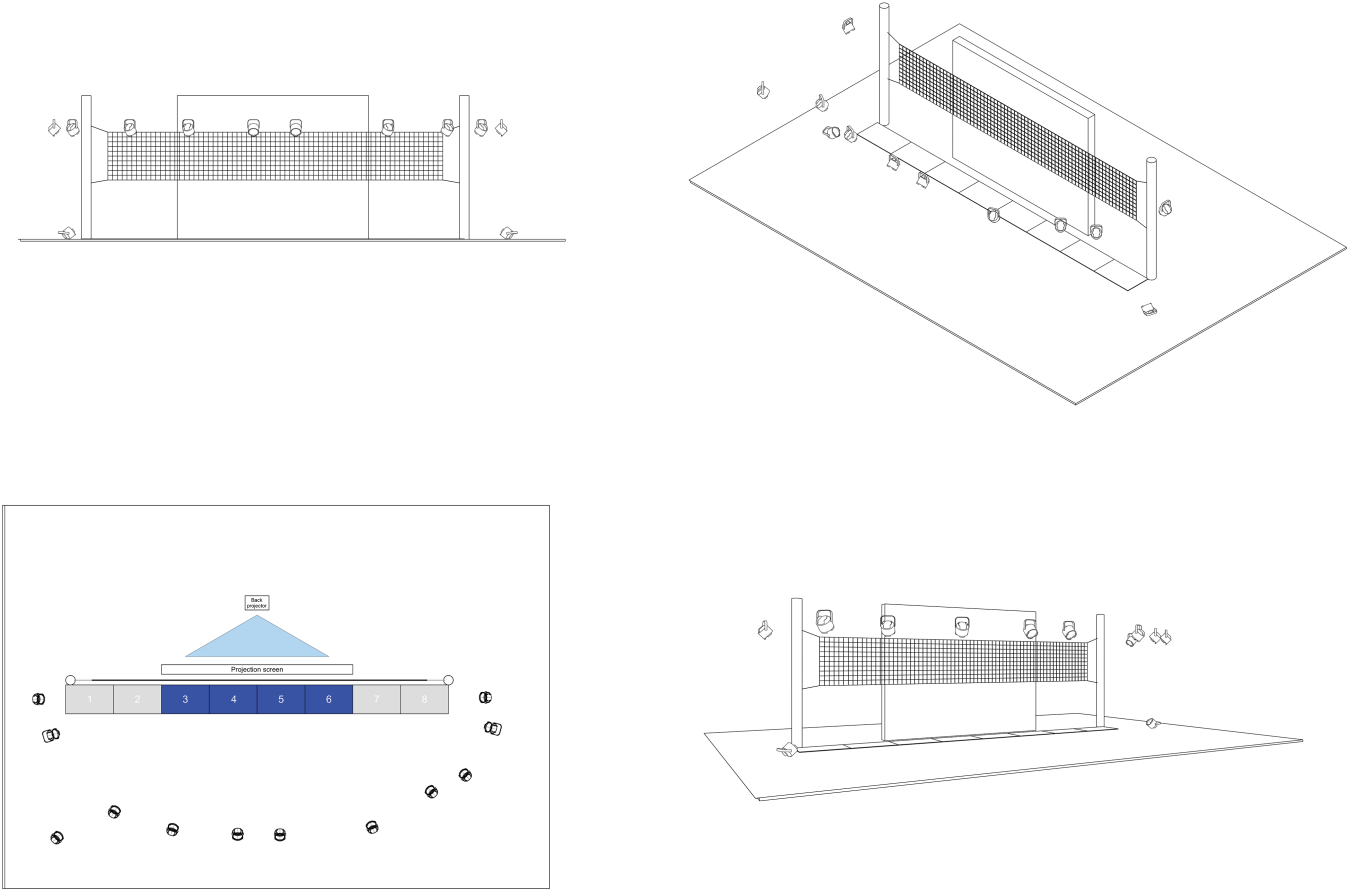
Experimental setup of the far-transfer task (adapted from Fleddermann and Zentgraf, 2018, p. 4).

The far-transfer task consisted of a volleyball-specific block decision task with different perceptual--cognitive demands. The setting of this task is explained with more details in Fleddermann and Zentgraf (2018, p. 3) and is presented in Figure 1. The test site consisted of a height-adjustable, standard volleyball net construction (9 m) parallel to a 5 m × 4 m projection screen placed in the middle of a motor behavior lab. To measure the jumping height, force plates (eight force plates, size: 60 cm × 80 cm, 1,200 Hz; Kistler^®^) and the Qualysis Track Manager (12 QTM Oqus cameras, 400 Hz, Qualisys^®^ version 2.15) motion capture system were synchronized, set around the net construction, and used for each measurement. The stimuli that were projected on the screen were presented with the Neurobehavioral System (NBS) Presentation^®^ software and synchronized with the QTM and Kistler systems.

In this setting, participants performed block jumps under three different conditions: a single-task (e.g., executing maximal isolated block jumps without a second perceptual-cognitive task) and two dual-task conditions [“dual-task low” (DT-L) and “dual-task high” (DT-H)]. In the “dual-task low” condition, a perceptual task in form of a picture of attackers was added, whereas in the DT-H condition, a dynamic video showed ball reception, set play, and attacking on either the left or the right side of the court:

1. *Single task (ST):* In the single task, athletes were instructed to execute isolated maximal block jumps to the right and left sides. Starting position was in front of the net; the screen in front of the net was gray; and the block jumps were self-initiated.
2. *Dual-task low (DT-L):* In the DT-L, athletes were instructed to execute maximal block jumps (to the left and right sides) according to a volleyball-specific static picture, which was presented on the screen in front of the net. The picture showed a freeze frame of an opponent’s offensive set play, which consisted of an attacking player, defense player, and a setter. Two pictures (for the left and right sides) were created with a GoPro^®^ Hero. Starting position was again in front of the net, and block jumps were self-initiated and executed in an individual technique and in maximal jumping height. Further, athletes were instructed to jump in front of the attacker on the picture. A static (perceptual) stimulus had to be processed without any time pressure or decision-making.
3. *Dual-task high (DT-H):* In the DT-H, athletes were instructed to perform maximal block jumps to the right and left sides depending on a volleyball-specific video (60 Hz), which was presented on the screen in front of the net. The stimuli, which were created with a GoPro^®^ Hero, showed an offensive set play (attacker, setter, and defense player) always with the same structure (reception, set play, and attack from position II or IV) from the perspective of a block player. The instruction for the participants was to observe the scene peripherally, decide to make a blocking action to the left or right side (depending on the attacker in the video), and to execute a maximal blocking action in front of the attacking player in the video. A dynamic, quickly changing stimulus had to be processed with the selection and execution of an adequate, maximal block action.

The setting, starting area (in the middle of force plates 4 and 5), and landing area (force plate 3 or 6) of the players were identical in each test. Each blocking trial was recorded in the QTM motion capture system (Version 2.15) and further processed by using MATLAB (Mathworks^®^, Version R2017a). Jumping height was calculated with MATLAB (Mathworks^®^, Version R2017a). Further parameters [volleyball-specific errors (e.g., net touching) and decision accuracy] were recorded by the experimenter *via* protocol. An invalid trial in decision accuracy was defined as a participant’s step in the wrong direction. All invalid trials were removed.

All conditions are described in more detail in Fleddermann and Zentgraf (2018).

### Statistical Analyses

To assess differences, repeated-measures ANOVAs were used. In the task-specific test (3D-MOT task) a 2 × 2 ANOVA with the factors group (intervention and control group) and test time (pre and post) were used. The average of the two sessions (analyzed with Microsoft Excel Version 16.10) in pre and post were used for the analysis. $\eta_{p}^{2}$ was used as a measure of effect size, and the level of significance was at *p* < 0.05.

For the near-transfer tests (letter readout, memory span, sustained attention, and processing speed), 2 × 2 ANOVAs with the factors group (intervention and control group) and test time (pre and post) were computed to assess differences. $\eta_{p}^{2}$ was used as a measure of effect size, and the level of significance was at *p* < 0.05.

A 2 × 2 × 3 ANOVA with the factors group (intervention and control group), test time (pre and post), and conditions [ST (single task), DT-L, and DT-H)] were computed to assess differences in the dependent variable jumping performance. $\eta_{p}^{2}$ was used as a measure of effect size, and the level of significance was at *p* < 0.05. Pairwise comparison with Bonferroni adjustments was used for the *post hoc* analysis. Invalid trials (a step in the wrong direction) were not analyzed. Data of each condition and participant were averaged for analysis with Microsoft Excel Version 16.10 and were analyzed with IBM SPSS statistics 25.

## Results

### Task-Specific Practice Test: 3D Mot

**Figure 2:**
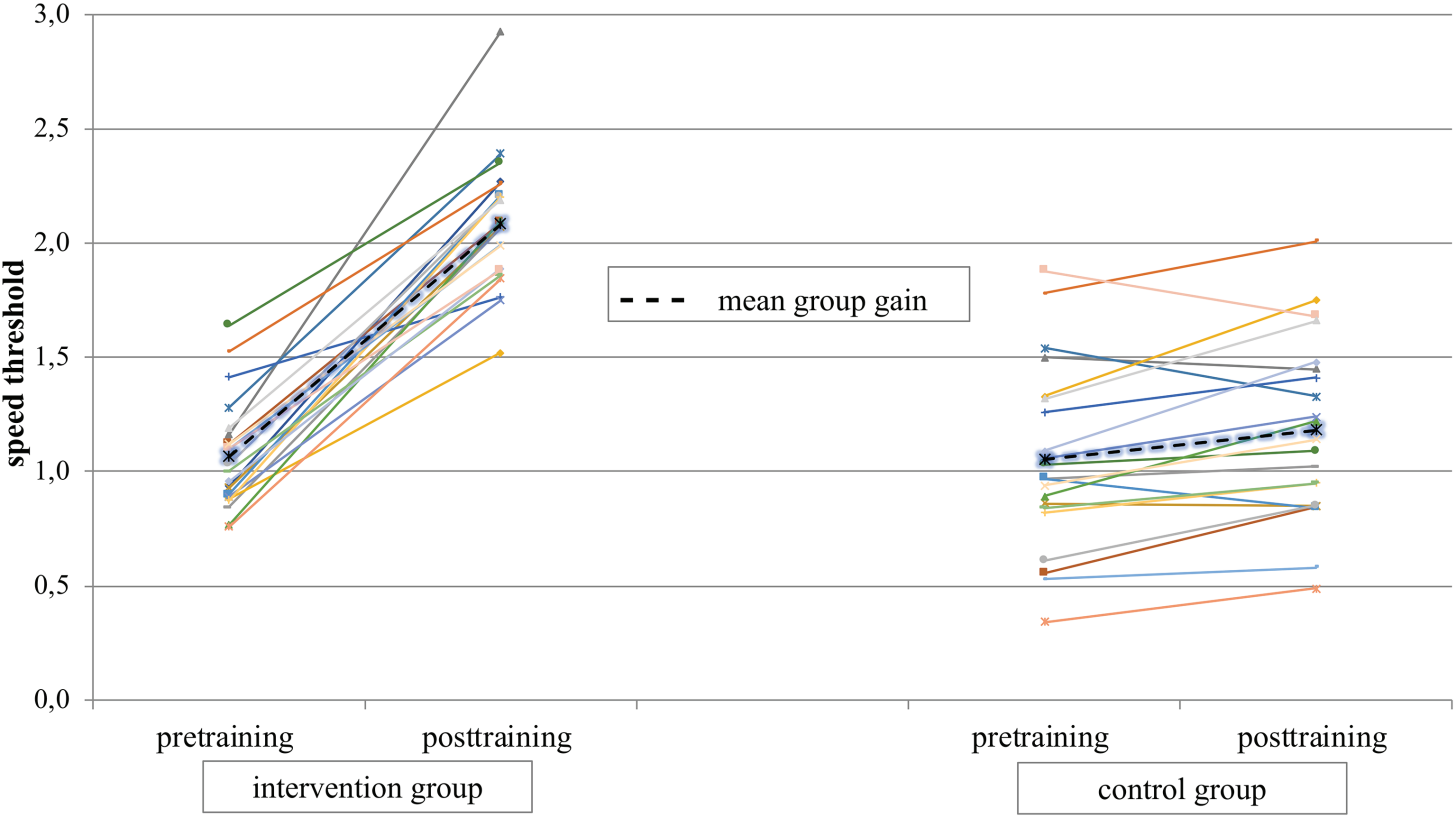
Mean speed threshold over all athletes for test time pre and post are presented in dashed lines. Individual data of athletes are presented in continuous lines.

Threshold speed of each participant was calculated on two sessions (20 trials). The mean average of the pretest was 1.05 (SD *=* 0.23) in the intervention group and 1.06 (SD *=* 0.40) in the control group. In the posttest, the results of the intervention group were 2.08 (SD *=* 0.29) and 1.18 (SD *=* 0.40) in the control group. Figure 2 shows the individual data of the intervention and control group for test time pre and post. The mean results for both groups are presented in dashed lines. The results of the 2 × 2 ANOVA with the factors group (intervention group/control group) and test time (pretraining/posttraining) showed a main effect for test time [*F*(1,40) = 162.75; *p* < 0.001, $\eta_{p}^{2}$= 0.80] and a significant interaction effect group*test time [*F*(1,40) = 93.10; *p* < 0.001; $\eta_{p}^{2}$ = 0.70].

### Near-Transfer Tasks

Three athletes of the control group were excluded from the data analysis in memory span and letter readout tests because of language problems (the tests could only be accomplished in German or in English, and the athletes did not speak sufficient German or English). Results of all near-transfer tests (intervention/control group, pre/posttest) are presented in Table 1.

**Table 1:** Mean results of all near-transfer tests (pre- and posttest) for both groups.

	Intervention group				Control group			
	Pretest		Posttest		Pretest		Posttest	
	*M*	SD	*M*	SD	*M*	SD	*M*	SD
Processing speed in s	56.23	(8.24)	46.8	(6.97)	57.06	(14.8)	51.81	(12.11)
Concentration ability score	180.91	(30.95)	224.23	(32.25)	177.52	(37.11)	201.57	(37.91)
Letter readout in s	5.16	(0.95)	4.89	(0.7)	5.4	(0.83)	5.49	(0.75)
Memory span score	6.15	(0.92)	6.55	(0.86)	6.11	(0.91)	6.06	(0.82)

**Memory Span:** Memory span was calculated as the sum of correct repetitions. The mean average of the intervention group was 6.15 (SD *=* 0.92) in pre and 6.55 (SD *=* 0.86) in post. The control group achieved a mean average of 6.11 (SD *=* 0.91) in pre and 6.06 (SD *=* 0.82) in post. The results of the 2 × 2 ANOVA of memory span with the factors group (intervention group/control group) and test time (pretraining/posttraining) showed no significant main effect for test time *F*(1,38) = 2.28; *p* = 0.14; $\eta_{p}^{2}$ = 0.06 and no significant interaction effect group*test time [*F*(1,38) = 2.27; *p* = 0.054; $\eta_{p}^{2}$ = 0.09].

**Letter Readout:** In the pretest, the mean reading time of the intervention group was 5.16 s (SD *=* 0.95) and 4.89 s (SD *=* 0.70) in the posttest. The results of the control group were 5.40 s (SD *=* 0.83) and 5.49 s (SD *=* 0.75) in pre and post. The results of the 2 × 2 ANOVA of memory span with the factors group (intervention group/control group) and test time (pretraining/posttraining) showed no significant main effect for test time [*F*(1,38) = 1.03; *p* = 0.316; $\eta_{p}^{2}$ = 0.04] and no significant interaction effect group*test time [*F*(1,38) = 2.01; *p* = 0.17; $\eta_{p}^{2}$ = 0.05].

**Figure 3:**
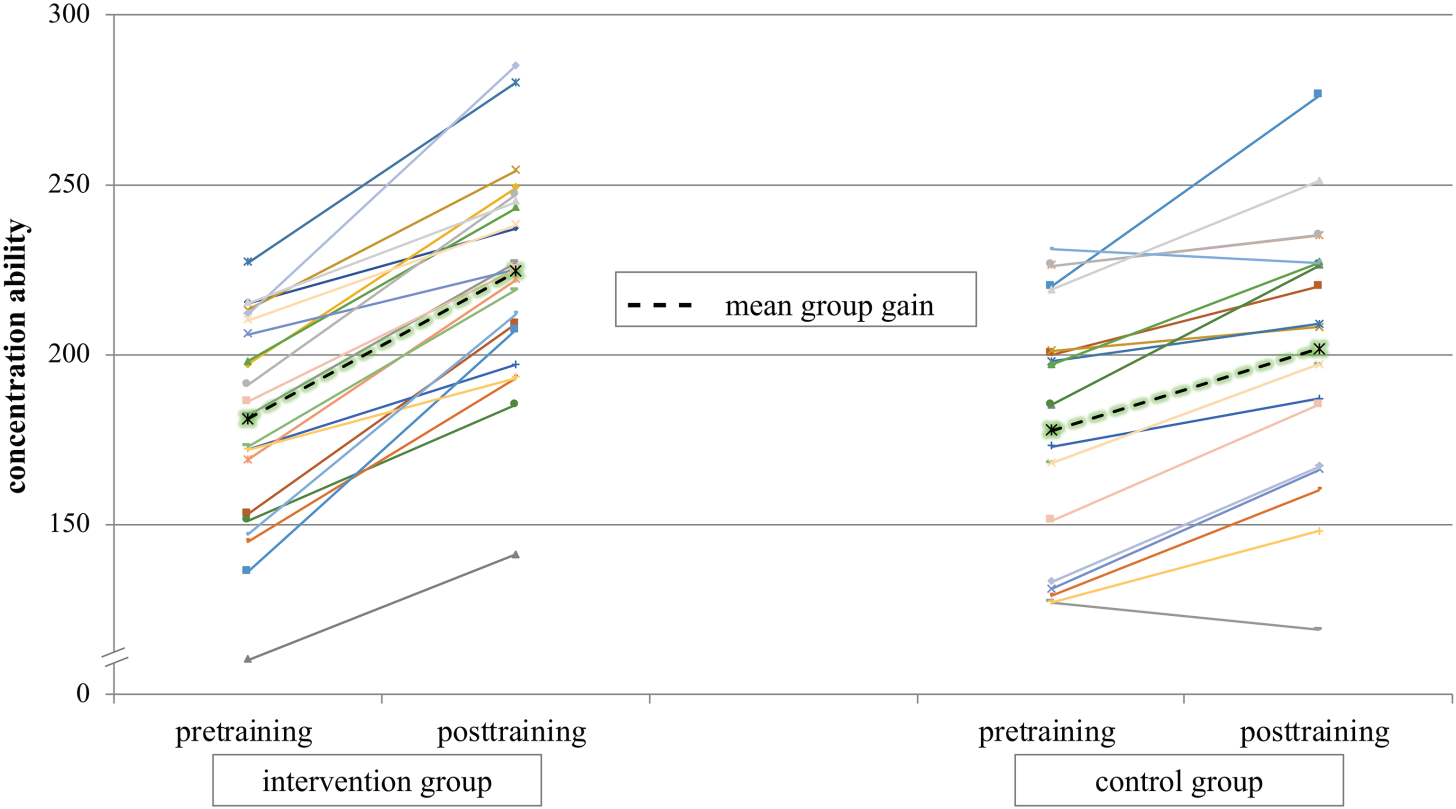
Mean concentration ability over all athletes for test time pre and post are presented in dashed lines. Individual data of athletes are presented in continuous lines.

**Sustained Attention:** Concentration ability was calculated based on processed target objects and errors. Results in the pretest were 180.91 (SD = 30.95) for the intervention group and 177.52 (SD = 37.11) for the control group. In the posttest, the intervention group achieved 224.23 (SD = 32.25) and the control group 201.57 (SD = 37.91). Figure 3 shows the individual data of the intervention and control groups for pretraining and posttraining. The mean results for both groups are presented as dashed lines. The results of the 2 × 2 ANOVA of sustained attention with the factors group (intervention group/control group) and test time (pre-training/post-training) showed a significant main effect for test time [*F*(1,40) = 182.78; *p* < 0.001; $\eta_{p}^{2}$ = 0.82] and a significant interaction effect group*test time [*F*(1,40) = 15.46; *p* < 0.001; $\eta_{p}^{2}$ = 0.28].

**Figure 4:**
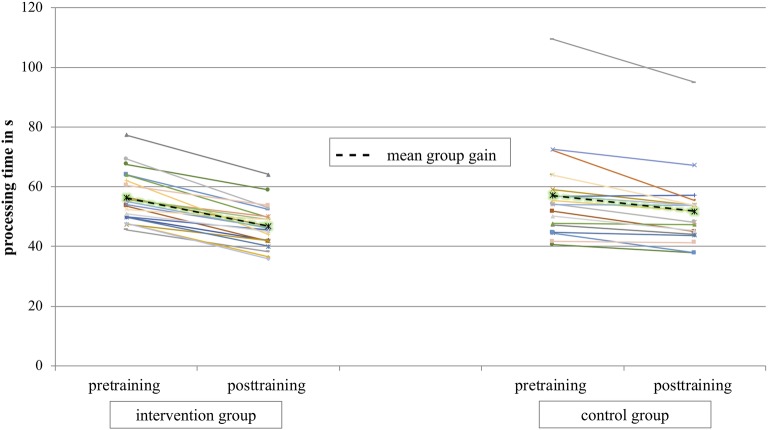
Mean processing speed time in seconds over all athletes for test time pre and post are presented in dashed lines. Individual data of athletes are presented in continuous lines.

**Processing Speed:** The intervention group achieved a mean processing speed time in pre of 56.23 s (SD = 8.24) and in post a mean processing time of 46.80 s (SD = 6.97). The result of the control group was 57.06 s (SD = 14.88) in pretest and 51.81 (SD = 12.11) in the posttest. Figure 4 shows the individual data of the intervention and control group for pretraining and posttraining. The mean results for both groups are presented as dashed lines. The results of the 2 × 2 ANOVA of processing speed with the factors group (intervention group/control group) and test time (pretraining/posttraining) showed a significant main effect for test time [*F*(1,40) = 124.87; *p* < 0.001; $\eta_{p}^{2}$ = 0.76] and a significant interaction effect group*test time [*F*(1,40) = 12.15; *p* = 0.001; $\eta_{p}^{2}$ = 0.23].

### Far Transfer

In the far-transfer tests, three participants of the control group were excluded from data analysis because of an injury or too many changes in the volleyball-specific blocking technique.

The response accuracy of the athletes in the intervention group was 96.9% in the pretest and 98.3% in the posttest. The control group achieved an average of 97.4% in the pretest and 98.2% in the posttest.

### Jumping Height

**Figure 5:**
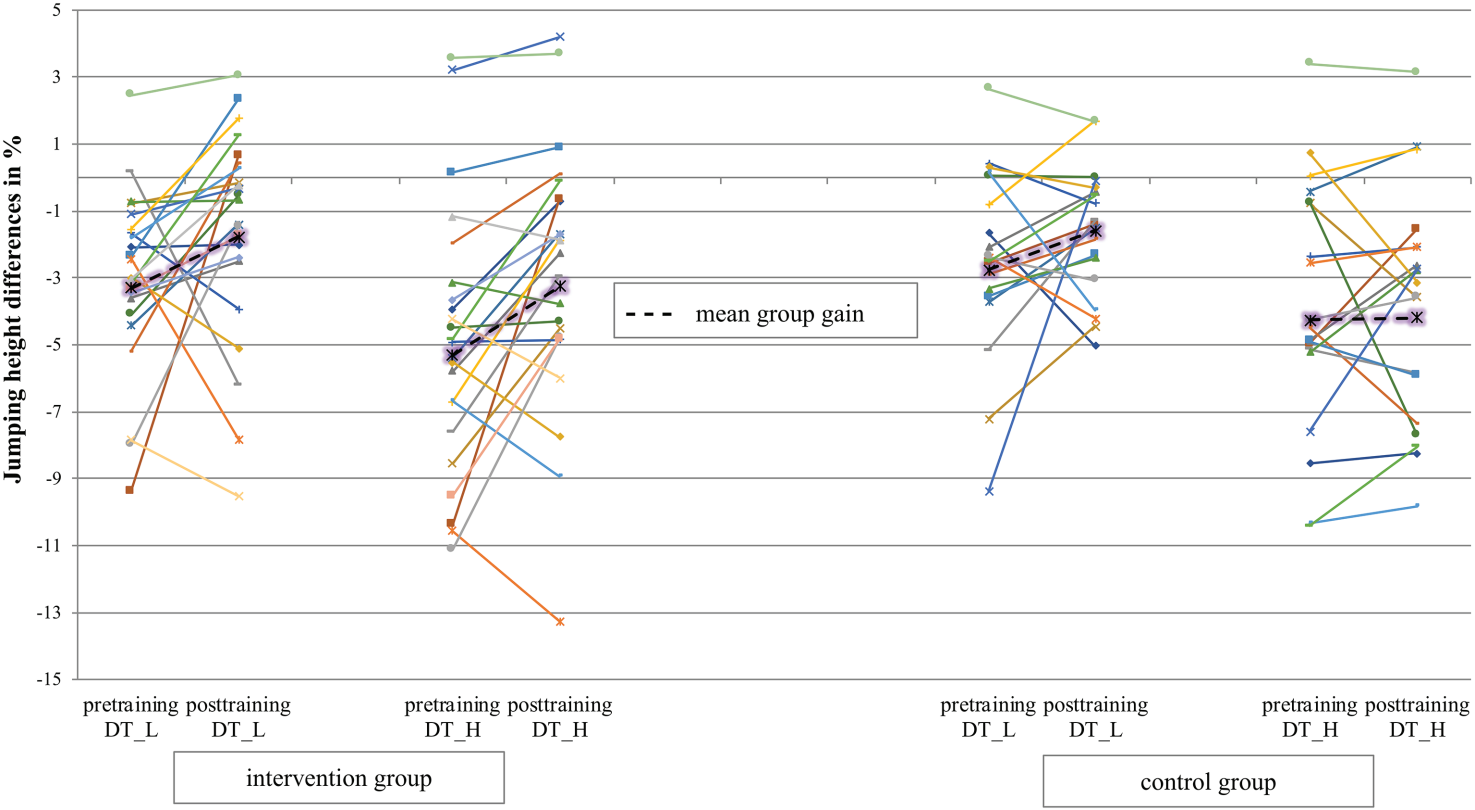
Mean percentage jumping height differences (from single-task to DT-H) over all athletes for test time pre and post are presented in dashed lines. Individual data of athletes are presented in continuous lines.

The mean jumping height was calculated based on individual means of each participant and each condition. Mean jumping performance of the intervention group was 48.85 cm in ST (SD = 7.62), 47.16 cm in DT-L (SD *=* 6.98), and 46.26 cm in DT-H (SD *=* 7.42) in the pretests and 46.9 cm in ST (SD = 7.78), 46.03 cm in DT-L (SD = 7.60), and 45.37 cm in DT-H (SD = 7.70). The mean jumping height of the control group was 48.66 cm in ST (SD = 7.01), 47.36 cm in DT-L (SD = 7.09), and 46.48 cm in DT-H (SD = 6.10) in pre and 48.65 cm in ST (SD = 7.2), 47.76 cm in DT-L (SD = 7.09), and 46.57 cm in DT-H (SD = 6.78). The jumping height differences in DT-L and DT-H were calculated based on single-task jumping height (100%) of each participant. In the pretest, participants in the intervention group jumped 5.31% (SD = 3.56) lower in the DT-H condition and 3.31% in the DT-L (SD = 2.45) condition. In the pretest, the control group jumped 4.27% (DT-H; SD = 3.40) and 2.76% (DT-L; SD = 2.65) lower. In the posttest, the intervention group jumped 3.26% (DT-H; SD = 3.70) and 1.79% (DT-L; SD = 3.06) lower and the control group 4.18% (DT-H; SD = 3.13) and 1.58% (DT-L; SD = 1.67). Mean and individual data (pre/post) of all athletes presented in Figure 5 (differences between ST and DT-L and differences between ST and DT-H). The results of the 2 × 2 × 3 ANOVA of jumping height differences with the factors group (intervention group/control group) and test time (pretraining/posttraining) and condition (single-task/DT-H/DT-L) show a significant main effect for condition [*F*(1,38) = 137.11; *p* < 0.001; $\eta_{p}^{2}$ = 0.28] and test time [*F*(1,38) = 51.95; *p* = 0.014; $\eta_{p}^{2}$ = 0.15] and no significant interaction effect group*test*condition [*F*(1,38) = 1.63; *p* = 0.21; $\eta_{p}^{2}$ = 0.04]. *Post hoc* analysis showed that ST was significantly higher than DT-L (*p* = 0.012) and DT-H (*p* = 0.01) in pre as well as in post (DT-L, *p* = 0.04 and DT-H, *p* = 0.01).

#### Discussion

The aim of the present study was to investigate the efficiency of an 8-week, generic off-court, visually based PCT in young elite volleyball players on three different levels: task-specific effects, near-transfer, and far-transfer effects. Based on the previous literature, we expected task-specific practice improvements as well as positive transfer effects (near/far) in the intervention group compared to the control group.

In line with our hypothesis, results showed that the intervention group performed significantly better in the task-specific practice test (3D-MOT) as well as in two near-transfer tests (sustained attention and processing speed) compared to the control group. The intervention group improved by 102% (3D-MOT), 24% (sustained attention), and 27% (processing speed) on average from pre to post. In the control group, improvements of 16% (3D-MOT), 14% (sustained attention), and 10% (processing speed) were observed. Contrary to our hypothesis, there were no significant differences in the other two near-transfer tests (letter readout and memory span test) between the intervention and control groups. Besides, in the lab-based blocking test (far-transfer test), no significant differences between the intervention and control groups were detected. At both test times (pre/post), the intervention and control groups showed a decreased jumping performance in the blocking task when a perceptual-cognitive load was added (dual tasks) compared to a blocking task without any additional stimuli (single task).

The findings of the study are in line with previous studies that analyzed task-specific practice effects and (near/far) transfer effects after a PCT in different populations as well as in the few studies in elite sports. Task-specific practice improvements were demonstrated in most intervention studies, e.g., healthy individuals or patients (Shipsted et al., 2012; Ballesteros et al., 2018). Further, studies in sport beginners or semiathletes (Larkin et al., 2015) also showed benefits in the trained task after a PCT. The few studies in elite sports mostly indicated (60%) task-specific practice effects (Zentgraf et al., 2017). Results imply that cognitive capacity is malleable *via* training interventions and that even elite athletes benefit from generic cognitive training interventions with improvements in the trained task. As we have only tested one single specific far-transfer task in our lab, we cannot rule out that there are other benefits of the intervention, e.g., better vigilance in practice and in games, higher binding due to variable practice, etc. Athletes in the intervention group improved on an individual level between 25 and 152% after the 16 sessions in the 3D-MOT task, which is similar to the results of Faubert (2013), who demonstrated improvements after 15 sessions in the 3D-MOT task with greatest learning rates for elite athletes (compared to semi- or nonathletes). Faubert (2013) explained this higher learning rates as a result of athletes having extraordinary skills for processing and learning an unpredictable, complex, visual tracking task.

Besides task-specific practice effects, main focus was to examine the transfer to tasks that were not directly trained. Most studies in elite sports did not address these levels (near/far) of transfer, even though it is an important question for researchers, athletes, and coaches. Results of this study regarding the near-transfer level show inconsistent findings. Indeed, near-transfer effects were shown in processing speed and sustained attention, but there were no effects in the two working memory tests. In contrast to our findings, Parsons et al. (2016) demonstrated improvements in a working memory test besides those in processing speed and in attention tests, suggesting cognitive skill transfer into untrained cognitive domains. In other populations such as adults, children, or the elderly (Shipsted et al., 2012) and other cognitive training tools, studies addressed near-transfer effects as well and found weak to moderate evidence for a near-transfer of cognitive training (Karbach and Verhaeghen, 2014). They also suggested a positive transfer based on overlapping cognitive processes between the practice task and the cognitive (near-transfer) tests. In the elite sport context, only a few studies examined near-transfer effects resulting in inconsistent findings (strong evidence versus no evidence). For example, Memmert and Schwab (2012) found no near-transfer effects after a 6-week visually based training, and also, Farrow and Abernethy (2002) detected no near-transfer effects after a temporal occlusion training over 4 weeks. However, Alsharji and Wade (2016) demonstrated evidence for near-transfer intervention effects after a perceptual training over seven sessions in elite athletes. However, in principle, there are too few studies in elite sports that investigated near-transfer effects. Overall, based on findings in studies of other domains, a transfer based on a high number of brain networks such as complex motion integration, distributed attention, processing speed, or working memory, which was required by executing PCT, seems possible.

Regarding the highest level of transfer–the transfer to the field (far-transfer)–Zentgraf et al. (2017) showed that only three studies in elite sports addressed this level, although this is of enormous importance for elite athletes and coaches. In game situations, it is indispensable for elite athletes’ success to interact quickly, for example, with teammates, opponents, or ball movements, and tap the full potential in (cognitive-)motor performance. Fleddermann and Zentgraf (2018) demonstrated that elite athletes show motor-cognitive interference in a dual-task situation using a game-like blocking lab task. The jumping height decreased when a perceptual-cognitive task (dual-task) was added compared to a self-initiated block jump without any additional load. Based on the theory of Wickens (2002), motor-cognitive interference was explained by overlapping resources between motor and perceptual-cognitive requirements, which led to a lower motor performance. The idea of this study was to reduce dual-task costs by a PCT intervention. As mentioned, the 3D-MOT task presumably recruits a high number of brain networks (e.g., sustained and distributed attention among others) and makes use of components that an athlete might also engage in a game situation. Therefore, we expected positive transfer from this off-court training task [combined with the sports-(un)specific movements] and the lab-based blocking task. However, results showed no significant differences (from pre to post) in jumping height between the intervention and control groups for the two dual-task conditions compared to the single task. Even if we did not find any significant differences in jumping height, a transfer could still have taken place at other (motor performance) parameters, e.g., reduced response or starting time. This underscores the difficulty of measuring far-transfer in a game-like sport situation. Sport situations are highly variable, and it is difficult to determine precisely which variables could be affected (Walton et al., 2018). Results of Romeas et al. (2016) also support this. After a 3D-MOT training intervention, they found improvements in passing accuracy only, but none in shooting or dribbling skills. From a theoretical perspective, the nature of the specific transfer was and is hard to predict. Furthermore, elite athletes already perform on a high level, and only small differences in jumping height might therefore be a meaningful change. Previous studies, which examined far-transfer effects in elite athletes, showed inconsistent findings regarding far-transfer effects. For example, Balasaheb and Sandhu (2008) showed improvements in batting performance within competition after a 6-week PCT. In contrast, Gorman and Farrow (2009) found no effects after a temporal-occlusion decision-making training in skilled basketball players in game decision accuracy. Mixed results further emphasize the difficulties in measuring far-transfer effects in a real-game sport situation. Furthermore, Hadlow et al. (2018) suggested to conduct further studies in the PCT area to address some main points (response correspondence, targeted perceptual function, and stimulus correspondence) to maximize the transfer into the field. Also, we did not specifically measure whether athletes improved in their peripheral visual field perception after the 3D-MOT training. This could be addressed in further studies. On the other side, the seminal aspect is the transfer to game situations. In this study, we hypothesized training-based reduction of motor-cognitive interference.

Furthermore, looking at the individual data of the intervention group, participants varied strongly in their results in the far-transfer task. Some participants benefited from improvements up to 6%, while other participants remained at their starting level. Besides that, most athletes in the intervention group were national youth players–individual factors might have influenced the outcome of the PCT. So, more studies in elite (youth) athletes are needed to investigate why some athletes benefit more than others and which theory can explain the underlying mechanism of (far-)transfer.

## Conclusion

The effectiveness of an off-court PCT and the effects of transfer especially to the field in elite sports are rarely investigated at the moment. So, this study is one of only a few that investigated different levels (of transfer) after an off-court PCT intervention in elite athletes. Overall, results suggested that cognitive capacity is malleable and improved after an additional off-court training. Results showed benefits of a PCT in task-specific practice tasks as well as in some near-transfer (processing speed/sustained attention) tasks. However, no significant transfer effects were shown in a game-like practice situation (far-transfer), which is the most important level in elite sports. So, the question how PCT is beneficial for different levels of transfer (for example to reduce cognitive-motor interference) is still open.

## Data Availability

The datasets generated for this study are available on request to the corresponding author.

## Ethics Statement

This study was carried out in accordance with the recommendations of University of Münster with written informed consent from all subjects. All subjects gave written informed consent in accordance with the Declaration of Helsinki. The protocol was approved by the University of Münster.

## Author Contributions

M-TF prepared the setup together with KZ, collected the data from the participants, analyzed the data, and wrote the manuscript. KZ was a grant applicant, developed the research design, supported setup preparation, checked the data, and wrote the manuscript. HH collected the data and checked the manuscript.

### Conflict of Interest Statement

The authors declare that the research was conducted in the absence of any commercial or financial relationships that could be construed as a potential conflict of interest.
